# A Comprehensive Disproportionality Analysis of Drug‐Related Head Injury Reports Using the FAERS Database

**DOI:** 10.1002/brb3.71549

**Published:** 2026-06-14

**Authors:** Jiaqi Guo, Luming Wei, Junfeng Liu, Jimei Zhang, He Wang, Wanjun Lu

**Affiliations:** ^1^ Department of Orthopedics The Affiliated Hospital of Beihua University Jilin City China; ^2^ Department of Oral Implantology The Affiliated Stomatological Hospital of Xuzhou Medical University Xuzhou China; ^3^ Department of Neurosurgery Tonghua Central Hospital Tonghua China; ^4^ Department of Hepatobiliary and Pancreatic Surgery Tonghua Central Hospital Tonghua China; ^5^ Department of Neurology The Affiliated Hospital of Beihua University Jilin City China; ^6^ Department of Neurology Jiangdu People's Hospital Affiliated to Yangzhou University Yangzhou China

**Keywords:** disproportionality analysis, FAERS database, head injury, pharmacovigilance, potential risks

## Abstract

**Objective:**

This study aimed to identify and characterize drugs associated with reports of head injury through a comprehensive analysis of the FDA Adverse Event Reporting System (FAERS) database.

**Methods:**

A retrospective disproportionality analysis was conducted on FAERS reports from 2004 to 2023. Cases were identified using a single Medical Dictionary for Regulatory Activities (MedDRA) Preferred Term: “Head Injury” (PT code 10019196). Four disproportionality methods—Reporting Odds Ratio (ROR), Proportional Reporting Ratio (PRR), Bayesian Confidence Propagation Neural Network (BCPNN), and Multi‐item Gamma Poisson Shrinker (MGPS)—were used to detect significant safety signals.

**Results:**

Analysis of 26,485 head injury reports revealed a rising trend over time, with 84.12% being serious and 8.51% fatal. Strongest signals were detected for coagulation factors, notably vonicog alfa (ROR: 43.69) and pegylated factor VIII (ROR: 26.93). Chamomile also showed a significant association (ROR: 20.10). In contrast, warfarin had the highest report count (*n* = 429) but only a moderate signal strength (ROR: 6.77).

**Conclusion:**

This large‐scale pharmacovigilance study identifies a strong association between coagulation factor therapies and reports of head injury. This signal most plausibly reflects confounding by indication rather than a direct prothrombotic mechanism. It also raises safety concerns regarding chamomile. These findings highlight the need for increased clinical vigilance and further investigation into these potential risks.

## Graphical abstract

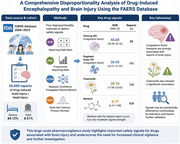

## 1 Introduction

Head injury, as defined by the Medical Dictionary for Regulatory Activities (MedDRA) preferred term (PT) “head injury” (PT 10019196), refers to traumatic damage to the scalp, skull, or intracranial contents resulting from external force. In the context of this pharmacovigilance study, we analyze spontaneous reports of head injury that may be temporally associated with drug exposure. It is important to note that the MedDRA term “head injury” predominantly captures traumatic events, and our analysis does not distinguish between traumatic and nontraumatic etiologies. Its clinical manifestations are diverse, ranging from mild cognitive impairment and confusion to severe seizures, coma, and even death, making it a serious and potentially disabling adverse event in clinical practice (Maas et al. 2017; Menon et al. 2010). As a severe adverse event that can lead to cognitive dysfunction, neurological deficits, or death, brain injury has complex and diverse etiologies, among which drug use is a significant trigger (L.‐Y. Zhang et al. 2025). This type of brain tissue damage, directly or indirectly induced by pharmacological treatment, is termed drug‐related head injury. Although not common in clinical practice, its consequences are severe, making it a critical concern in drug safety monitoring. Drug‐related head injury can be caused by various medications, including but not limited to antineoplastic agents, immunosuppressants, antimicrobials, and central nervous system drugs (Salama et al. 2022; Bailly 2019). Although clinical trials evaluate drug safety before market approval, factors such as limited sample sizes, short follow‐up durations, and selection bias in study populations make it difficult to fully identify certain rare or delayed adverse reactions—such as brain injury—prior to commercialization (D. Li et al. 2023).

Post‐marketing pharmacovigilance is essential for identifying potential adverse drug reactions (Roldan Munoz et al. 2023). The United States FDA Adverse Event Reporting System (FAERS) is an open, spontaneous reporting database that collects adverse drug event reports from around the world, providing a valuable resource for large‐scale, real‐world drug safety signal detection. In recent years, studies using FAERS have successfully identified neurotoxicity signals associated with specific drugs (P. Chen et al. 2023). However, a systematic comprehensive analysis focusing specifically on drug‐related head injury is still lacking.

This study aims to mine FAERS data from 2004 to 2023 using disproportionality analysis methods to comprehensively assess drug signals associated with brain injury, with particular attention to those drugs not currently labeled with such risks, thereby providing valuable insights for clinical medication safety.

## 2 Methods

### 2.1 Data Source

This retrospective analysis was based on data extracted from the FAERS database (https://www.fda.gov/Drugs/GuidanceComplianceRegulatoryInformation/Surveillance/AdverseDrugEffects/ucm082193.htm; Oliveira et al. 2025). This system contains voluntarily submitted reports from healthcare professionals and consumers worldwide, including detailed information such as patient demographics (DEMO), drug information (DRUG), adverse event terms (REAC), outcomes (OUTC), report sources (RPSR), and indications for use (INDI) (Zhao et al. 2025). Data from the first quarter of 2004 to the fourth quarter of 2023 were included to enable systematic capture of long‐term drug safety signals. To ensure data quality and minimize the impact of duplicate reports, we applied the standard FDA‐recommended deduplication strategy. Specifically, for all reports sharing the same CASEID, we retained only the most recent version based on the FDA_DT (FDA receipt date) and CASEID (case identifier), removing all older submissions.

### 2.2 Identification of Adverse Events and Medicinal Products

Adverse events were coded using PTs from the MedDRA (Version 24.0) (Garmann et al. 2025). In this study, we used a single PT—“head injury” (MedDRA code: 10019196)—to identify cases of interest. All reports containing this PT from the first quarter of 2004 to the fourth quarter of 2023 were downloaded. We acknowledge that this PT primarily captures traumatic head injuries (e.g., from falls or accidents) as defined by MedDRA. Therefore, our analysis reflects associations between drugs and reports of head injury rather than nontraumatic, drug‐related head injury. This definitional limitation is addressed further in the Sections 4 and 6. Reports containing agents not listed in the DrugBank database (https://go.drugbank.com/drugs) were considered invalid and excluded (Wishart et al. 2018).

### 2.3 Data Analysis Methods

Descriptive analysis was used to summarize the clinical characteristics of patients with brain injury, including age, gender, indication, outcome, and reporting country. To enhance the specificity of the signal and minimize confounding from concomitant medications, the disproportionality analysis was restricted to reports where the drug of interest was listed as the “Primary Suspect (PS)” using the FAERS ROLE_COD variable. The frequency of reports for each drug associated with brain injury was counted, and the top 20 drugs by signal intensity were selected for further analysis.

Four disproportionality analysis methods were employed to evaluate the strength of association between drugs and brain injury (Arku et al. 2022):

1. *Reporting Odds Ratio (ROR)*: This method assesses whether the proportion of reports for a target drug‐event pair (i.e., a specific drug and brain injury) is significantly disproportionate compared to the proportion for all other drugs and brain injury (Ooba and Kubota 2010). Calculation involves a 2 × 2 contingency table (Table 1), where: “*a*” = number of reports of brain injury with the target drug, “*b*” = number of reports of all other adverse events with the target drug, “*c*” = number of reports of brain injury with all other drugs, “*d*” = number of reports of all other adverse events with all other drugs.
2. *Proportional Reporting Ratio (PRR)*: This algorithm analyzes whether the proportion of brain injury reports for the target drug among all its reports is significantly higher than the proportion of brain injury reports for all other drugs among their reports (Evans et al. 2001).
3. *Bayesian Confidence Propagation Neural Network (BCPNN)*: This method uses a neural network model to explore complex relationships between the target drug and brain injury in large datasets, measuring the association strength via the information component (IC) (Yang et al. 2020).
4. *Multi‐Item Gamma Poisson Shrinker (MGPS)*: This empirical Bayesian method accounts for data uncertainty and variability (Trippe et al. 2017). The Empirical Bayes Geometric Mean (EBGM) and its lower 95% confidence interval (EBGM05) are calculated.

**TABLE 1:** Ratio imbalance measurement algorithm.

Item	Reports with the brain injury	All other AEs	Total
Reports with the target drug	*a*	*b*	*a* + *b*
All other drugs	*c*	*d*	*c* + *d*
Total	*a* + *c*	*b* + *d*	*a* + *b* + *c* + *d*

The specific calculation formulas and criteria for these four methods are summarized in Table 2.

**TABLE 2:** Principle of disproportionality measure and standard of signal detection.

Algorithms	Calculation formula	Criteria
ROR	$\mathrm{ROR}=\frac{a/c}{b/d}=\frac{ad}{bc}$ $95\%\mathrm{CI}=e^{\ln\left(\mathrm{ROR}\right)\pm 1.96\sqrt{\frac{1}{a}+\frac{1}{b}+\frac{1}{c}+\frac{1}{d}}}$	(1) *a* ≥ 3 (2) ROR ≥ 2 (3) 95% CI > 1
PRR	$\mathrm{PRR}=\frac{a/(a+b)}{c/(c+d)}=\frac{a(c+d)}{c(a+b)}$ $\chi^{2}=\frac{{\left(\left|ad-bc\right|-\frac{n}{2}\right)}^{2}n}{\left(a+b)(a+c)(c+d)(b+d\right)}$ *n* = *a* + *b* + *c* + *d*	(1)a≥3 (2) PRR ≥ 2 (3)$\chi^{2}\geq 4$
BCPNN	$E\left(\mathrm{IC}\right)=\log_{2}\frac{\left(C_{xy}+\gamma_{11}\right)\left(C+\alpha \right)\left(C+\beta \right)}{\left(C+\gamma \right)\left(C_{x}+\alpha_{1}\right)\left(C_{y}+\beta_{1}\right)}$	(1) *a* ≥ 3 (2) IC‐2SD > 0
	$V\left(\mathrm{IC}\right)=\frac{1}{{\left(\ln2\right)}^{2}}\left\{\left(\frac{C-C_{xy}+\gamma -\gamma_{11}}{\left(C_{xy}+\gamma_{11}\right)\left(1+C+\gamma \right)}\right)+\left(\frac{C-C_{x}+\alpha -\alpha_{1}}{\left(C_{x}+\alpha_{1}\right)\left(1+C+\alpha \right)}\right)+\left(\frac{C-C_{y}+\beta -\beta_{1}}{\left(C_{y}+\beta_{1}\right)\left(1+C+\beta \right)}\right)\right\}$ $\gamma =\gamma_{11}\frac{\left(C+\alpha )(C+\beta \right)}{\left(C_{x}+\alpha_{1}\right)\left(C_{y}+\beta_{1}\right)}$ $\mathrm{IC}-2\mathrm{SD}=E\left(\mathrm{IC}\right)-2\sqrt{V(\mathrm{IC})}$ $\alpha_{1}=\beta_{1}=1;\alpha =\beta =2;\gamma_{11}=1;$ $C=a+b+c+d;C_{x}=a+b;C_{y}=a+c;C_{xy}=a$	

Abbreviations: BCPNN, Bayesian confidence propagation neural network; CI, confidence interval; IC, information component; PRR, proportional reporting ratio; ROR, reporting odds ratio.

*Time‐to‐onset analysis*: For drugs with sufficient report volume, we attempted to estimate the time from drug start to head injury event. Only reports with documented drug start date and event date were included. The time‐to‐onset was calculated as the difference between these dates (in days). Given the expected high rate of missing data, this analysis was considered exploratory with limited reliability. All data extraction, cleaning, and disproportionality analyses were performed using R software (Version 4.3.2). Signal detection was primarily conducted using the FAERS R package, and visualization was performed with the ggplot2 package.

## 3 Results

### 3.1 Descriptive Analysis

**FIGURE 1:**
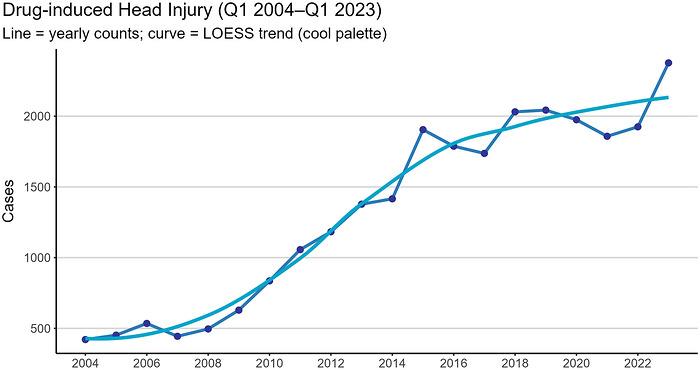
Number of reported cases of drug‐related head injury from Q1 2004 to Q4 2023. The bar chart shows the total number of unique reports containing the MedDRA preferred term "head injury" each year. A total of 26,485 reports were included.

A total of 26,485 unique reports associated with drug‐related head injury were ultimately included in the analysis. As shown in Figure 1, the number of brain injury‐related reports generally showed an increasing annual trend, rising from 421 (1.59%) in 2004 to 2377 (8.97%) in 2023, with the most concentrated reporting period occurring between 2015 and 2023. The clinical characteristics of these 26,485 reports are presented in Table 3. Regarding demographics, female patients slightly outnumbered males (56.98% vs. 38.33%). The highest proportion of reports was among elderly patients aged ≥ 75 years (20.66%), followed by those aged 45–65 (22.60%) and 65–75 (15.10%), suggesting a possible increase in risk with age. Concerning report sources, over half of the reports were submitted by consumers (50.47%), followed by physicians (20.02%) and pharmacists (11.44%). Geographically, over half of the reports originated from the United States (50.90%), with the remainder primarily from Canada (5.39%), Italy (3.04%), and the United Kingdom (2.39%). In terms of clinical outcomes, the vast majority of reports (84.12%) were classified as serious adverse events. The most common outcomes were “other serious events” (42.80%) and “hospitalization or prolonged hospitalization” (41.32%). Furthermore, 8.51% of reports resulted in patient death, and 3.59% were life‐threatening events.

**TABLE 3:** Clinical characteristics of reported drug‐related head injury.

Variable	Total
Sex	
Female	15,090 (56.98)
Male	10,151 (38.33)
Unknown	1244 (4.70)
Age (year)	
< 19	841 (3.18)
19–45	2644 (9.98)
45–65	5985 (22.60)
65–75	4000 (15.10)
≥ 75	5471 (20.66)
Unknown	7544 (28.48)
Reporter	
Consumer	13,366 (50.47)
Physician	5303 (20.02)
Pharmacist	3030 (11.44)
Other health‐professional	2915 (11.01)
Unknown	1600 (6.04)
Lawyer	254 (0.96)
Registered nurse	17 (0.06)
Reported countries	
United States	13,480 (50.90)
Other	7454 (28.14)
Canada	1427 (5.39)
Italy	805 (3.04)
United Kingdom	634 (2.39)
France	618 (2.33)
Brazil	451 (1.70)
Japan	260 (0.98)
Germany	235 (0.89)
Australia	213 (0.80)
Colombia	164 (0.62)
Argentina	139 (0.52)
Spain	119 (0.45)
Israel	102 (0.39)
Outcomes	
Other serious	14,491 (42.80)
Hospitalization	13,990 (41.32)
Death	2881 (8.51)
Life threatening	1215 (3.59)
Disability	1022 (3.02)
Required intervention to prevent permanent impairment/damage	200 (0.59)
Congenital anomaly	61 (0.18)

**FIGURE 2:**
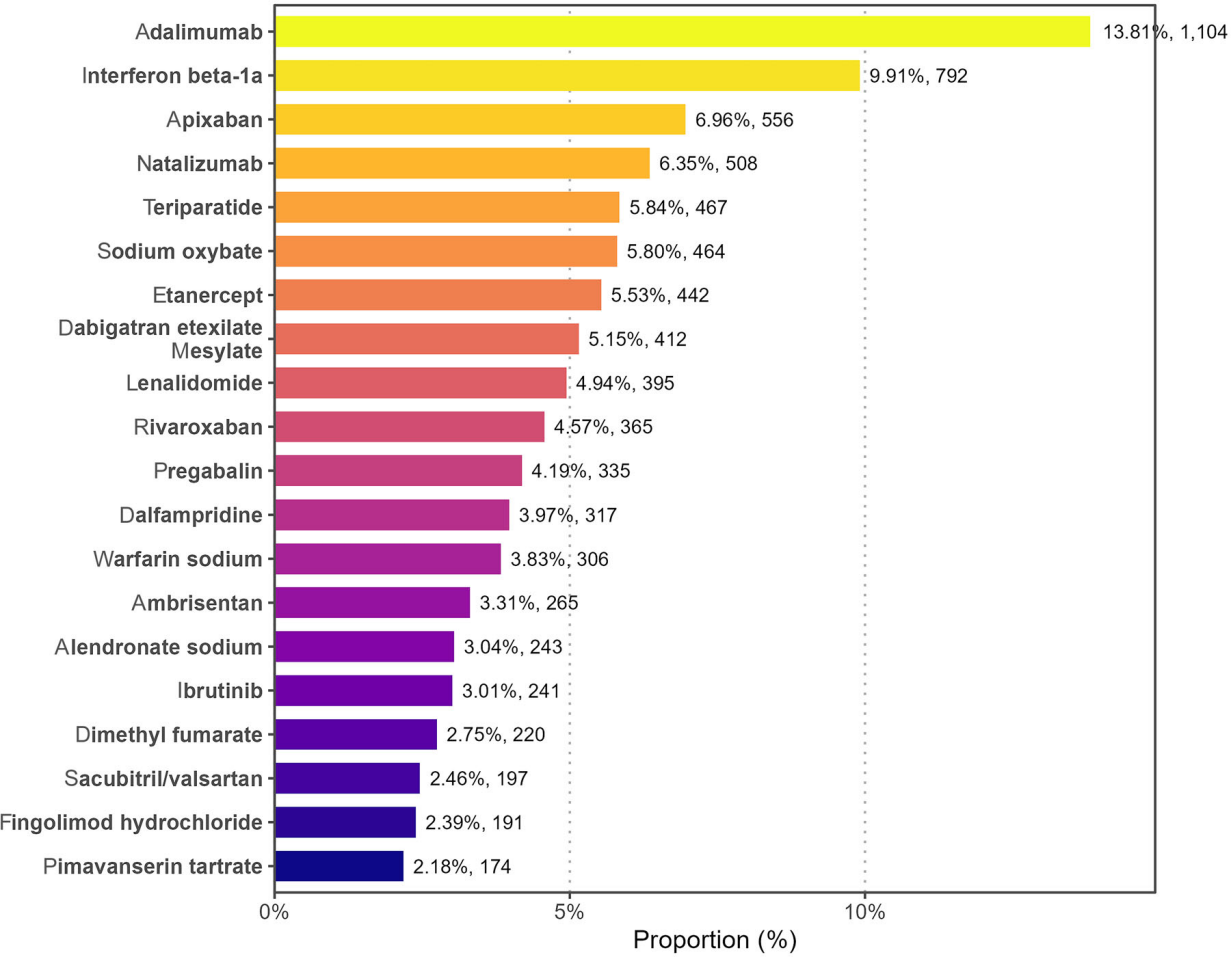
Top 20 drugs with the highest number of reported head injuries. The chart displays the proportion of head injury reports attributed to each drug among all reports where the drug was listed as the primary suspect (PS).

A frequency analysis of the reported drugs listed the top 20 drugs most frequently associated with brain injury reports (Figure 2). The most frequently reported drugs were primarily biologics, led by adalimumab, and anticoagulants. Adalimumab (a biologic, 1104 reports, 13.81%) had the highest count, followed by interferon beta‐1a (792 reports, 9.91%) and the anticoagulant apixaban (556 reports, 6.96%). In addition, drugs for multiple sclerosis (e.g., natalizumab, dimethyl fumarate, fingolimod), anticoagulants (e.g., rivaroxaban, dabigatran etexilate, warfarin sodium), and neurological agents (e.g., sodium oxybate, pregabalin) were also frequently reported.

### 3.2 Association Analysis Between Drugs and the Target Adverse Event

We identified a total of 987 brain injury signals, ranked in descending order based on the four disproportionality analysis methods (Supporting Information). Table 4 lists the top 20 drugs with the most significant disproportionality signals for the target adverse event, ranked by ROR and PRR point estimates. vonicog alfa yielded the strongest signal, with an ROR of 43.69 (95% CI: 13.58–140.59), a PRR of 41.02 (95% CI: 13.69–122.94), and an IC of 5.36 (IC025 = 3.89). Pegylated coagulation factor VIII (ROR: 26.93, 95% CI: 11.90–60.94), and chamomile (ROR: 20.10, 95% CI: 8.26–48.95) also exhibited remarkably strong signal strengths, ranking second and third, respectively. We attempted a preliminary time‐to‐onset analysis. However, onset dates and dosage information were highly incomplete: over 85% of reports lacked usable information on either drug start date or dosage. Among the minority of reports with usable start dates (*n* = 3219), the median time‐to‐onset was approximately 17 days (interquartile range: 7–44 days). Due to the extremely high proportion of missing data, these estimates are of limited reliability and should be interpreted with caution.

**TABLE 4:** Top 20 drugs for signal strength.

Drug	Case reports	ROR (95% CI)	PRR (95% CI)	χ^2^	IC (IC025)	EBGM (EBGM05)	Labeling status
Vonicog alfa	3	43.69 (13.58, 140.59)	41.02 (13.69, 122.94)	117.3	5.36 (3.89)	41.02 (15.43)	Potential new signal
Pegylated coagulation Factor VIII	6	26.93 (11.9, 60.94)	25.91 (11.83, 56.75)	143.88	4.7 (3.6)	25.91 (13.08)	Potential new signal
Chamomile	5	20.1 (8.26, 48.95)	19.54 (8.25, 46.29)	88.05	4.29 (3.11)	19.53 (9.28)	Potential new signal
Emicizumab	136	18.01 (15.19, 21.37)	17.56 (15.01, 20.54)	2116.43	4.13 (3.88)	17.48 (15.15)	Labeled
Antihemophilic factor VIII	77	17.92 (14.29, 22.48)	17.47 (14.08, 21.67)	1194.16	4.12 (3.8)	17.43 (14.41)	Potential new signal
Eftrenonacog alfa	41	17.31 (12.69, 23.61)	16.89 (12.59, 22.66)	613.07	4.08 (3.63)	16.87 (13.01)	Potential new signal
Antihemophilic factor, human recombinant	221	15.71 (13.74, 17.96)	15.37 (13.4, 17.63)	2948.31	3.93 (3.74)	15.25 (13.63)	Potential new signal
Safinamide	5	14.56 (6, 35.33)	14.27 (6.02, 33.8)	61.78	3.83 (2.67)	14.27 (6.8)	Labeled
Coagulation factor IX	53	13.89 (10.58, 18.24)	13.62 (10.35, 17.92)	619.59	3.77 (3.38)	13.6 (10.83)	Potential new signal
Terazosin	17	10.12 (6.27, 16.35)	9.98 (6.24, 15.97)	137.57	3.32 (2.65)	9.98 (6.68)	Labeled
Stiripentol	11	8.67 (4.78, 15.71)	8.57 (4.76, 15.43)	73.6	3.1 (2.28)	8.56 (5.21)	Labeled
Aducanumab	6	8.44 (3.77, 18.88)	8.34 (3.73, 18.63)	38.83	3.06 (1.98)	8.34 (4.25)	Labeled
Isosorbide mononitrate	13	7.85 (4.54, 13.56)	7.77 (4.49, 13.45)	76.71	2.96 (2.2)	7.76 (4.91)	Labeled
Lacosamide	148	7.34 (6.24, 8.63)	7.27 (6.21, 8.5)	796.96	2.85 (2.62)	7.23 (6.31)	Labeled
Isoptin	3	7.2 (2.31, 22.47)	7.13 (2.33, 21.79)	15.84	2.83 (1.41)	7.13 (2.75)	Labeled
Gliadel	5	6.9 (2.86, 16.65)	6.84 (2.89, 16.2)	24.95	2.77 (1.61)	6.84 (3.27)	Labeled
Gabitril	3	6.85 (2.2, 21.37)	6.79 (2.22, 20.75)	14.83	2.76 (1.34)	6.79 (2.62)	Potential new signal
Bevacizumab (rhumab vegf)	5	6.81 (2.82, 16.44)	6.75 (2.85, 15.99)	24.54	2.76 (1.59)	6.75 (3.23)	Potential new signal
Human c1‐esterase inhibitor	3	6.78 (2.17, 21.14)	6.72 (2.2, 20.54)	14.63	2.75 (1.32)	6.72 (2.59)	Potential new signal
Warfarin	429	6.77 (6.15, 7.45)	6.71 (6.08, 7.4)	2053.4	2.73 (2.59)	6.62 (6.11)	Labeled

*Note*: All disproportionality signals represent statistical associations detected in FAERS. For coagulation factor products, these signals may be confounded by the underlying bleeding disorder (confounding by indication) and should not be interpreted as novel drug‑specific risks without further validation.

Notably, a distinct cluster of drugs related to hemophilia management and coagulation disorders was prominent among the top‐ranked signals. This included emicizumab (ROR: 18.01, 95% CI: 15.19–21.37; *n* = 136 reports), antihemophilic factor VIII (ROR: 17.92, 95% CI: 14.29–22.48), eftrenonacog alfa (ROR: 17.31, 95% CI: 12.69–23.61), and recombinant human antihemophilic factor (ROR: 15.71, 95% CI: 13.74–17.96, *n* = 221 reports). All these drugs demonstrated consistent and robust signals across all four analytical methods, reinforcing their potential association. Another noteworthy finding was that although warfarin had the highest number of reported cases (*n* = 429) among these 20 drugs, its signal strength was relatively moderate (ROR: 6.77, 95% CI: 6.15–7.45). This indicates that the relationship between warfarin and this adverse event is well‐established and frequently reported, but its disproportionality is significantly lower compared to the strongest signals mentioned above.

## 4 Discussion

Previously, J. Li et al. (2024) analyzed drug‐induced hypoglycemia using FAERS data. Meanwhile, W. Chen and Chen (2025) conducted a safety analysis of amantadine, a drug used for treating brain injuries. This study presents the first comprehensive disproportionality analysis of drug‐related head injury using large‐scale FAERS data. We systematically delineated the clinical characteristics of patients reported with these events and, more importantly, identified a series of drugs with strong signals, revealing a significant risk cluster centered around coagulation factors. These findings provide novel and crucial insights into the epidemiological features and potential mechanisms of drug‐related head injury.

First, our descriptive analysis underscores the severity and clinical burden of drug‐related head injury. The existence of over 26,000 reports, coupled with an increasing annual trend, indicates that this is not a rare clinical issue. Particularly alarming is the finding that 84.12% of reports were classified as serious adverse events, with 8.51% resulting in patient death. This aligns with the understanding that drug‐related head injury has severe consequences, highlighting its priority in post‐marketing drug safety monitoring. The age distribution, showing patients ≥ 65 years as the highest risk group, correlates closely with the pathophysiological characteristics of this population, including declined hepatic and renal function, multimorbidity, polypharmacy, and reduced blood–brain barrier integrity, rendering them more susceptible to drug‐induced neurotoxicity (Malagaris et al. 2020).

The core finding of this study is the strong statistical association between coagulation factor products—specifically vonicog alfa, pegylated factor VIII, and emicizumab—and reports of head injury. However, interpreting this signal requires careful consideration of the underlying patient population. These drugs are primarily indicated for patients with severe bleeding disorders, including von Willebrand disease and hemophilia A/B (Cau et al. 2024). This population has an inherently high baseline risk of intracranial hemorrhage due to their coagulopathy, independent of any drug therapy (Kim et al. 2022). Spontaneous or trauma‐induced intracranial bleeding is a well‐recognized complication of severe hemophilia, particularly when factor replacement is suboptimal (Doshi et al. 2024).

Therefore, the disproportionality signal likely reflects confounding by indication—that is, the natural history of the underlying disease—rather than a direct neurotoxic or prothrombotic effect of the drugs themselves. For example, a patient with severe hemophilia who experiences a fall (which may be more frequent due to hemophilic arthropathy or physical limitations) can develop a head injury with intracranial bleeding that is subsequently reported as an adverse event. In the FAERS database, such an event may be attributed to the drug (the factor product) even if the drug was appropriately administered. While some thrombotic complications have been reported with certain factor products—most notably emicizumab when used concomitantly with bypassing agents—the majority of head injury events in this population are likely hemorrhagic rather than ischemic (Carcao et al. 2019; K. Zhang et al. 2023). Our analysis, which relies on a single MedDRA PT “head injury,” cannot distinguish between hemorrhagic and thrombotic etiologies.

Consequently, we caution against interpreting these signals as evidence of a prothrombotic mechanism. Instead, the signals highlight an important clinical reality: Patients receiving coagulation factor therapies remain at significant risk for head injury—particularly from trauma or spontaneous bleeding—and require close monitoring, fall prevention strategies, and prompt evaluation of any neurological symptoms. Future studies using granular clinical data (including imaging findings and cause of head injury) and more specific case definitions (such as distinguishing intracranial hemorrhage from ischemic stroke) are needed to clarify whether these signals represent drug‐attributable risk, disease‐related risk, or a combination of both.

Another insightful finding was the signal associated with the herbal agent chamomile (ranked third by ROR). Commonly perceived by the public as a safe and harmless soothing remedy (Howrey et al. 2016), our analysis identifies a signal that merits further investigation. However, this finding is based on a small number of reports (*n* = 5) and may be influenced by confounding by indication or reporting bias. Therefore, these results should be considered hypothesis‑generating only, and no firm conclusions about causality or clinical risk can be drawn at this stage. While clinical data are scarce, preclinical pharmacological studies provide plausible mechanisms that could warrant exploration, including the presence of coumarin derivatives with anticoagulant properties, modulation of GABA‑A receptors by flavonoids, and potential immunomodulatory effects (Czigle et al. 2023; Cerri et al. 2017). Nonetheless, these mechanistic hypotheses require confirmation in dedicated studies. This signal suggests that herbal and dietary supplements warrant continued safety monitoring similar to prescription drugs, but confirmatory studies with larger sample sizes and better confounder control are needed.

The case of warfarin provides an excellent paradigm for understanding the nature of FAERS data. Despite having the highest absolute number of reports (*n* = 429), its disproportionality signal strength (ROR = 6.77) was relatively modest. This illustrates a key principle of disproportionality analysis: widely used drugs with well‐known adverse event profiles have high background reporting rates, which naturally lowers the disproportionality metric. Warfarin exemplifies this principle—its moderate ROR does not imply a lower absolute risk; rather, it reflects the high baseline frequency of reporting for its well‐established association with hemorrhagic head injury (Chakraborty and Tiwari 2025; Tollefsen et al. 2018). In contrast, drugs with fewer total reports but much higher disproportionality, including vonicog alfa and other coagulation factors, may represent signals that are less recognized or associated with newer products.

Overall, these results should alert clinicians, researchers, and regulators to the underappreciated neurotoxic potential of hemostatic agents and certain herbal products. Future studies should aim to validate these signals using complementary data sources, elucidate the underlying molecular mechanisms, and develop risk mitigation strategies to protect vulnerable patients.

## 5 Conclusion

This study identified a strong statistical association between coagulation factor drugs and reports of head injury in the FAERS database. However, due to confounding by indication and the inability to distinguish hemorrhagic from thrombotic events, these signals should not be interpreted as evidence of a prothrombotic mechanism. Instead, they underscore the persistently high risk of head injury and intracranial bleeding in patients with severe bleeding disorders, even when receiving factor replacement therapy. Clinicians should remain vigilant for head trauma and bleeding complications in this vulnerable population, and future pharmacovigilance studies should employ more specific case definitions and adjust for disease severity to better isolate drug‐attributable risks.

## 6 Limitations

This study has several limitations:

1. *Case definition and data completeness limitations*: The most significant limitation of this study is our exclusive reliance on the single MedDRA PT “head injury” (PT 10019196) to define cases. This term primarily captures traumatic head injury (e.g., from falls or accidents) and does not directly capture nontraumatic conditions such as encephalopathy or metabolic brain injury. Therefore, our findings represent associations between drugs and reports of head injury, not drug‐related head injury in the pathophysiological sense. In addition, a major constraint for deeper mechanistic insight was the profound incompleteness of key data fields. Specifically, over 85% of reports lacked usable information on either drug start date or dosage, which severely limits any time‐to‐onset or dose‐response analysis. The median time‐to‐onset of 17 days, while reported, is based on a small and potentially biased subset of complete reports and should not be considered representative.
2. *Confounding by indication and lack of adjustment*: A core limitation of disproportionality analysis is its inability to effectively control for confounding variables. This is especially pertinent for drugs used in severe bleeding disorders (for instance, vonicog alfa and emicizumab), where the treated population has a high preexisting risk of intracranial hemorrhage independent of drug therapy. Patients with severe hemophilia or von Willebrand disease have an inherently higher risk of head injury and intracranial bleeding due to their underlying coagulopathy, as well as an increased fall risk from hemophilic arthropathy. Therefore, it is difficult to entirely disentangle to what extent the observed risk is caused by the drug itself versus the severity of the underlying condition—a classic example of confounding by indication. Moreover, our case definition does not differentiate between hemorrhagic and thrombotic events. The strong signals for coagulation factors are more plausibly explained by underlying coagulopathy and bleeding tendency rather than a direct drug‐induced prothrombotic effect. Future studies using more specific outcome definitions (such as “intracranial hemorrhage” vs. “ischemic stroke”) and adjusting for disease severity are urgently needed.
3. *Inability to establish causality*: This study identifies statistical associations, not causality. Disproportionality analysis is an excellent hypothesis‐generating tool, but its results must be validated through other study designs. Further research should be directed based on these findings.
4. *Lack of “Denominator Data”*: FAERS only provides numerator data (number of reports with the adverse event) but lacks denominator data (total number of exposed individuals). Therefore, it is impossible to calculate the true incidence rate of adverse events in the real world or to compare the absolute size of risks between different drugs.

## Author Contributions

**Junfeng Liu**: methodology. **Jiaqi Guo**: writing – original draft. **Jimei Zhang**: software. **Wanjun Lu**: writing – review and editing. **He Wang**: data curation. **Luming Wei**: conceptualization.

## Funding

This work was supported by Yangzhou City Basic Research Program (Joint Special Project) ‐ Health and Wellness Category (NO.2024‐2‐29) and Yangzhou City Science and Technology Project (NO.YZ2024061).

## Ethics Statement

The authors have nothing to report.

## Conflicts of Interest

The authors declare no conflicts of interest.

## Supporting information




**Supplementary Material**: brb371549‐sup‐0001‐SuppMat.xlsx

## Data Availability

The datasets generated and analyzed during the current study are available in the possession of the corresponding author and can be made available to any qualified researcher upon a formal request.
